# Advances in Analysis of Flavors and Fragrances: Chemistry, Properties and Applications in Food Quality Improvement

**DOI:** 10.3390/foods14111992

**Published:** 2025-06-05

**Authors:** Ana Leahu, Marìa Soledad Prats Moya, Cristina Ghinea

**Affiliations:** 1 Faculty of Food Engineering, Stefan cel Mare University of Suceava, 720229 Suceava, Romania; analeahu@fia.usv.ro; 2 Analytical Chemistry, Nutrition and Food Sciences Department, University of Alicante, San Vicente del Raspeig, 03690 Alicante, Spain

Natural flavors and fragrances are an exciting area for both academic and industrial research as consumer preference for natural and sustainable products continues to grow. Flavor influences the well-being of humans and is considered a multisensory experience that encompasses taste, smell, and texture [1]. Human chemical senses are stimulated by organic compounds that can be found in fruits, spices, teas, coffee, mushrooms, milk, meat, fermented products, and others [2].

The profiles of these compounds in foods are influenced by various factors, such as species, geographical origin, growing conditions, and processing methods [3]. Raw materials and production processes (malting, crushing, fermentation, distillation, and maturation) shape the flavor of spirits. Esters are formed during alcoholic fermentation and give these alcoholic beverages floral and fruity notes. Additionally, the barrel maturation process is another crucial phase in which the spirit develops depth, character, and a harmonious blend of flavors. Fruit variety, agronomic factors related to the orchard, processing technologies, fermentation, and maturation conditions, influences the sensory profiles and chemical quality of ciders. Wheat variety, geographical location, and harvest year can affect the grain composition and volatile composition of whisky. The cultivar, growing conditions, and post-harvest processing technologies (baked, roasted, steamed, and sundried tea) influence the final sensorial perception of the teas. Pollination, bagging, harvesting period, storage conditions, and calcium regulation influence the fruit’s aroma.

Flavor compounds in fruit are mainly organic acid esters, aldehydes, and volatile phenols, while alcohols, aldehydes, ketones, esters, and acids were identified in teas. Maillard reaction is responsible for meat products’ flavor, while fatty acids, carbonyl compounds, and methyl sulfide can contribute to the flavor of dairy products [4]. In alcoholic beverages, alcohols, acids, aldehydes, ketones, esters, ethers, hydrocarbons, pyrrolines, pyridines, furans, and sulfur compounds were identified [5]. Aldehydes, alcohols, ketones, furans, and pyrazines are key aroma compounds determined in soy, and their identification is crucial for improving the overall sensory acceptability of soy products [6].

The food industry and scientists are actively investigating the identification, isolation, and concentration of organic flavor compounds by various methods [7]. Aroma components can be isolated from foods mainly through stir bar sorptive extraction (SBSE) and solid-phase microextraction (SPME) techniques. Additionally, headspace (HS) is the best technical mode for visualizing volatile compounds’ profile [8]. The detection of volatile organic compounds (VOCs) can be performed with Gas Chromatography–Mass Spectrometry (GC-MS), Gas Chromatography–Ion Mobility Spectrometry (GC-IMS), Headspace Solid-Phase Microextraction Gas Chromatography–Mass Spectrometry (HS-SPME-GC-MS) techniques, electronic nose (EN), electronic tongues (ETs), colorimetric sensor array (CSA), and others [9]. The main drawback of some techniques is still volatile quantification, as there is a lack of commercial volatile standards.

The final assessment of a food’s flavor quality largely depends on human emotions and preferences when selecting and consuming it. Therefore, flavoring agents are commonly used to mask unpleasant flavors in food. Consumers associate health with natural additives, but artificial additives show higher sensory efficiency and flavor acceptance if the consumer has not consulted the product label. Therefore, it is crucial to understand the influence of neurocognitive mechanisms and perceptions on consumption decisions.

The primary aim of this Special Topic (ST), “Advances in Analysis of Flavors and Fragrances: Chemistry, Properties and Applications in Food Quality Improvement”, is to investigate recent advances and current knowledge in the study of flavor and fragrance in different types of foods and beverages. This ST comprises 36 papers which address subject-like investigations of the aromatic components of wines (Grenache and Huangjiu), liquors (Baijiu, Luzhou-flavored liquor, Hunan Light-Flavor Baijiu), plum brandies, apple juice and cider, Jerez vinegars, Irish whiskey, sun-dried green tea, raw Pu-erh tea, and baked green tea, green coffee, traditional fermented koumiss, Hurood cheese, cow, donkey, camel, and horse milk powder, chicken carcasses, fermented soybeans, soy sauce, and others products. The usability of electronic noise and tongue data and volatile compounds’ determination and finding the relationship between key metabolites and taste attributes are analyzed in different matrices and with the help of multivariate statistical methods (please see the list of contributions for further details). We believe that further investigations in this scientific field, including artificial intelligence (AI) and nanotechnology, will provide solutions for the food industry in terms of flavor and fragrance applications, as well as controlled release from food products.

## References

[1] Hossain M.S., Wazed M.A., Asha S., Hossen M.A., Fime S.N.M., Teeya S.T., Jenny L.Y., Dash D., Shimul I.M. (2025). Flavor and Well-Being: A Comprehensive Review of Food Choices, Nutrition, and Health Interactions. Food Sci. Nutr..

[2] Reshna K.R., Gopi S., Balakrishnan P., Balakrishnan P., Gopi S. (2022). Introduction to flavor and fragrance in food processing. Flavors and Fragrances in Food Processing: Preparation and Characterization Methods.

[3] Yang H., Wu M., Shen X., Lai Y., Wang D., Ma C., Ye X., Sun C., Cao J., Sun C. (2025). Comprehensive Review of VOCs as a Key Indicator in Food Authentication. eFood.

[4] Wei G., Dan M., Zhao G., Wang D. (2023). Recent advances in chromatography-mass spectrometry and electronic nose technology in food flavor analysis and detection. Food Chem..

[5] Wu J., Liu Y., Zhao H., Huang M., Sun Y., Zhang J., Sun B. (2021). Recent advances in the understanding of off-flavors in alcoholic beverages: Generation, regulation, and challenges. J. Food Compos. Anal..

[6] Xu J., Zeng M., Wang Z., Chen Q., Qin F., Chen J., He Z. (2025). Characteristic flavor compounds in soy protein isolate and their correlation with sensory attributes. Food Res. Int..

[7] Tananaki C., Liolios V., Kanelis D., Rodopoulou M.A. (2022). Investigation of Volatile Compounds in Combination with Multivariate Analysis for the Characterization of Monofloral Honeys. Appl. Sci..

[8] Starowicz M. (2021). Analysis of Volatiles in Food Products. Separations.

[9] Feng Y., Wang Y., Beykal B., Qiao M., Xiao Z., Luo Y. (2024). A mechanistic review on machine learning-supported detection and analysis of volatile organic compounds for food quality and safety. Trends Food Sci. Technol..

